# Family-based genome-wide association of inflammation biomarkers and fenofibrate treatment response in the GOLDN study

**DOI:** 10.1186/s12919-018-0146-5

**Published:** 2018-09-17

**Authors:** Sarmistha Das, Pronoy Kanti Mondal, Saurabh Ghosh, Indranil Mukhopadhyay

**Affiliations:** Human Genetics Unit, Indian Statistical Institute, 203 B T Road, Kolkata, 700108 India

## Abstract

In this paper we analyzed whole-genome genetic information provided by GAW20 from the Genetics of Lipid Lowering Drugs and Diet Network (GOLDN) study for family data. Lipid levels such as triglycerides (TGs) and high-density lipoprotein (HDL) are measured at different time points before and after administration of an anti-inflammatory drug fenofibrate. Apart from that, the data contain some covariates and whole-genome genotype information. We propose 2 novel approaches based on Henderson’s iterative mixed model to identify associated loci corresponding to (a) inflammatory biomarkers like TGs and HDLs together over time, and (b) the response to fenofibrate treatment. We developed a mixed-model approach using both TG and HDL phenotypes at all 4 time points for a genetic association study whereas we used TGs only to study genetic association with response to the drug. We expect that use of complete family data in a longitudinal framework under a single model involving the appropriate correlation structures would be able to exploit the maximum possible information contained in the sample. Our analysis of whole-genome single nucleotide polymorphisms (SNPs) and genomic regions corresponding to drug treatment finds no significant locus after multiple correction. Arguably, the moderately small sample size of the data set, as compared to the sample size usually used in genome-wide association studies (GWAS), could be a reason for such a result. Nevertheless, we report the top 20 SNPs associated with the phenotypes, and the top 20 SNPs and genomic regions associated with a response to fenofibrate treatment. Application of our methods to larger GWAS and further functional validation of the reported top SNPs and genomic regions might provide important biological insight into the genetic constitution of the trait.

## Background

Understanding the genetic architecture underlying complex phenotypes is crucial in decoding disease mechanisms as well as treatment and drug development. Genome-wide association studies (GWAS) have contributed significantly to the identification of associated variants with numerous traits. Although the sample size requirement of GWAS is high, the proportion of the disease risk explained by a single variant always remains low. However, availability of longitudinal data on multiple phenotypes might carry more information in identifying associated variants. The Genetics of Lipid Lowering Drugs and Diet Network (GOLDN) study provides a data set with triglyceride (TG) and high-density lipoprotein (HDL) levels at 4 time points for a fixed set of families with few missing observations during follow-up. Consequently, given a moderate sample size, reduction of the multiple testing burden and/or use of longitudinal information is required. Moreover, with available data on multiple interacting phenotypes, it is informative to study both the inherent environmental correlation and the genetic correlation.

Increased levels of TG and decreased HDL levels are well-known causes of heart disease. So we developed one model that captures the genome-wide genetic association with interacting phenotypes, that is, TG and HDL, over time by introducing both a temporal covariance structure and a genetic covariance structure between phenotype measurements. Our method is expected to increase power as a result of the inclusion of more information through genetic and environmental correlation structures.

On the other hand, fenofibrate is an antiinflammatory drug, well known for TG-lowering effects. Some studies report modulation of a lipid response to fenofibrate as the result of genetic variants involved in lipid metabolism [1], but the response to treatment by fenofibrate varies across individuals in a population [2]. Some GWAS ventured to find associated variants behind such a response, but met no success in the sense of identifying variants significantly associated with fenofibrate response [3]. The reasons might be low sample size, noncomparable baseline lipid profiles, and environmental exposures of the individuals. Thus, along with simple GWAS, we studied multiloci association of response to fenofibrate treatment with the genomic regions, which reduces the chance of missing a moderately associated locus. We also examined the association of single variants using multiple TG and HDL phenotypes in the GOLDN study. We found that 1 single nucleotide polymorphism (SNP) is associated with the TG and HDL phenotypes, although we did not find any significant SNP or gene that is associated with the drug response. It is important to note that because the sample in this study is not very large, we report a few top significant loci that might be associated with phenotype and response to drug.

## Methods

We analyzed the real data provided by GAW20 (ie, GOLDN study) data set [4]. The data set contains data on age, smoking status, etc. as covariates, pedigree information, and genome-wide genetic variation, as well as TG and HDL levels measured before and after the drug at 4 time points. Information on genetic variation was available for 822 individuals, while other information, except kinship structure, had a sample size of 1105 individuals. Pedigree data was available for 4151 individuals. The kinship structure, covariates, and TG and HDL phenotypes were available for all 822 individuals, but genotype information was missing for 1 individual. Consequently, in the subsequent analysis we used the remaining 821 subjects. Next, the missing genotypes and monomorphic SNPs were removed from the analysis. For variants with only 2 observed genotypes, we eliminated the SNP if 1 genotype frequency was < 5%. We imputed missing phenotype data using a mixed-model approach under the null model, and used log–log transformation of the phenotype variables for the entire analysis. This transformation made the data normally distributed and, hence, the resultant test statistic followed a standard distribution under a null hypothesis (H_**0**_) of no association. During imputation, we assumed constant heritability, and this value was from an existing study [5]. We calculated *p* values using the asymptotic distribution of test statistics under H_**0**_ after Benjamini-Hochberg (BH) correction.

To meet the objectives as stated in the previous section, we first do a GWAS based on longitudinal data with TG and HDL together as phenotypes. We use a mixed model that includes environmental as well as genetic correlation structure.

With TG and HDL at all time points as response vector, our model is:

1 $$ \left(\begin{array}{c}{Y}^{TG}\\ {}{Y}^{HDL}\end{array}\right)=\left(\begin{array}{c}{X}^{TG}{\beta}^{TG}+{Z}^{TG}{u}^{TG}\\ {}{X}^{HDL}{\beta}^{HDL}+{Z}^{HDL}{u}^{HDL}\end{array}\right)+\left(\begin{array}{c}{\varepsilon}^{TG}\\ {}{\varepsilon}^{HDL}\end{array}\right) $$

where ${Y}^{TG}={\left({Y}_1^{TG},{Y}_2^{TG},{Y}_3^{TG},{Y}_4^{TG}\right)}^{\hbox{'}}$ and ${Y}^{HDL}={\left({Y}_1^{HDL},{Y}_2^{HDL},{Y}_3^{HDL},{Y}_4^{HDL}\right)}^{\hbox{'}}$ denote TG and HDL respectively, at 4 time points, for *n* individuals. In this model, *X*^*TG*^ and *X*^*HDL*^ are fixed effects design matrices, where *X*^*TG*^ = *X*^*HDL*^ = [*I*_4_ ⊗ 1_*n*_  1_4_ ⊗ *g*_*n*_], *g*_*n*_ is the genotype vector for *n* individuals at a single marker locus, *u*^*TG*^ and *u*^*HDL*^are random effect vectors for *n* individuals, and *Z*^*TG*^ = *Z*^*HDL*^ = 1_4_ ⊗ *I*_*n*_ is the corresponding matrix. Here ${\beta}^{TG}={\left({\beta}_1^{TG},{\beta}_2^{TG},{\beta}_3^{TG},{\beta}_4^{TG},{\beta}_5^{TG}\right)}^{\hbox{'}}$, where the first 4 components are temporal effects for 4 different time points that includes the drug effect and ${\beta}_5^{TG}$ is the effect of the SNP. We assume that, $Var\left({u}^{TG}\right)={\sigma}_{u, TG}^2K, Var\left({u}^{HDL}\right)={\sigma}_{u, HDL}^2K$ where *K* is the kinship matrix, *Var*(*ε*^*TG*^) = Σ_*TG*_ ⊗ *I*_*n*_, *Var*(*ε*^*HDL*^) = Σ_*HDL*_ ⊗ *I*_*n*_,

$$ {\Sigma}_{TG}={\sigma}_{TG}^2\left(\begin{array}{cccc}1& {\rho}_{1, TG}& {\rho}_{1, TG}{\rho}_{2, TG}& {\rho}_{1, TG}{\rho}_{2, TG}{\rho}_{3, TG}\\ {}{\rho}_{1, TG}& 1& {\rho}_{2, TG}& {\rho}_{2, TG}{\rho}_{3, TG}\\ {}{\rho}_{1, TG}{\rho}_{2, TG}& {\rho}_{2, TG}& 1& {\rho}_{3, TG}\\ {}{\rho}_{1, TG}{\rho}_{2, TG}{\rho}_{3, TG}& {\rho}_{2, TG}{\rho}_{3, TG}& {\rho}_{3, TG}& 1\end{array}\right) $$

where ${\sigma}_{TG}^2$ is the common variance of TG at all 4 time points and the correlation coefficient matrix is parameterized by three parameters: *ρ*_1, *TG*_, *ρ*_2, *TG*_ and *ρ*_3, *TG*_. Similarly we define *β*^*TG*^ and Σ_*HDL*_. Now, denoting *ρ*_*g*_ and *ρ*_*ε *_as genetic and environmental correlations respectively, we assume the correlation structure as:

$$ Var\left(\begin{array}{c}{Y}^{TG}\\ {}{Y}^{HDL}\end{array}\right)=Z\left({\Sigma}_{u, TG, HDL}\otimes K\right){Z}^{\prime }+\left({\Sigma}_{\varepsilon, TG, HDL}\otimes {I}_n\right) $$ where $$ Z=\left(\begin{array}{cc}{Z}^{TG}& 0\\ {}0& {Z}^{HDL}\end{array}\right), $$ $$ {\Sigma}_{\varepsilon, TG, HDL}=\left(\begin{array}{cc}{\Sigma}_{TG}& {\rho}_{\varepsilon }{\Sigma}_{TG}^{\frac{1}{2}}{\Sigma}_{HDL}^{\frac{1}{2}}\\ {}{\rho}_{\varepsilon }{\Sigma}_{HDL}^{\frac{1}{2}}{\Sigma}_{TG}^{\frac{1}{2}}& {\Sigma}_{HDL}\end{array}\right), $$ $$ {\Sigma}_{u, TG, HDL}=\left(\begin{array}{cc}{\sigma}_{u, TG}^2& {\rho}_g{\sigma}_{u, TG}{\sigma}_{u, HDL}\\ {}{\rho}_g{\sigma}_{u, HDL}{\sigma}_{u, TG}& {\sigma}_{u, HDL}^2\end{array}\right). $$.

We test the null hypothesis of no genetic association of an SNP with TG and HDL using a likelihood ratio test. The asymptotic distribution of the log-likelihood ratio statistic can be shown to follow a ${\chi}_2^2$ distribution. We apply this test at the GWAS level after appropriate multiple-testing correction.

To address our second objective (ie, to test association of response to fenofibrate treatment), we model our data using Henderson’s mixed-model approach with adequate modification. Incorporating correlation structure among family members, we propose our model as

2 $$ Y= X\beta + Zu+\varepsilon $$

where *Y* is the vector of changes in phenotype (measured as log-log of TG) before and after the drug treatment; *X* is the design matrix of covariates, namely, age, smoking status, and SNP/genotypes in a genomic region (not in linkage disequilibrium); *β *is the associated fixed effect parameter; *Z* is the design matrix of random components; *u* is the random effect of the family; and *ε* is the error component. We assume $u\sim N\left(0,{\sigma}_g^2K\right)$, where *K* is the kinship matrix and $\varepsilon \sim N\left(0,{\sigma}_e^2R\right)$ independently of *u*. Hence, $V(Y)={\sigma}_g^2 ZK{Z}^{\prime }+{\sigma}_e^2R$. Note that because we are dealing with family data, a non-diagonal positive definite matrix *R* appears in the variance-covariance matrix of *ε*.

During analysis, we use 778 individuals after removing those with no response either before or after drug treatment. But in case of missing response at one of the time points before (after) drug treatment, we impute it with the other response value. To calculate kinship matrix we use R package “kinship2” with the entire family data. To find association of genomic regions, we first identify the genomic regions and then remove the SNPs that are in linkage disequilibrium (*r*^*2*^ > 0.5). The genomic regions are basically (a) the genic regions, and (b) the intergenic regions lying between 2 consecutive genes, that overlap the genotyped SNPs in our data. We use Henderson’s iterative procedure for mixed model approach [6] after substantial modification and after adjusting for age, smoking status as fixed effects, and random genetic effect within a family. We use the restricted maximum likelihood (REML) approach to test our *H*_*0*_ of no association, adopting the expectation-maximization (EM) algorithm for parameter estimation.

Maximization of joint likelihood of *Y* and *u* and eq. (3) [6] provide the best linear unbiased predictors (BLUPs) for the random component under normal assumption of the response variable.

3 $$ \left(\begin{array}{c}{X}^{\prime }{R}^{-1}X\widehat{\beta}+{X}^{\prime }{R}^{-1}Z\widehat{u}\\ {}{ZR}^{-1}X\widehat{\beta}+\left({Z}^{\prime }{R}^{-1}Z+{D}^{-1}\right)\widehat{u}\end{array}\right)=\left(\begin{array}{c}{X}^{\prime }{R}^{-1}y\\ {}{Z}^{\prime }{R}^{-1}y\end{array}\right) $$

So to test the association of (a) whole-genome SNPs and (b) genomic regions, with response to the drug treatment, our null hypothesis will be, *H*_0_ : *Mβ* = 0 for an arbitrary *p* × *q* matrix *M* with *rank*(*M*) = *p*. Thus, if *n* be the number of observations and *rank*(*M*) = *p*, the test statistic [7],

4 $$ F=\frac{{\left(M\widehat{\beta}\right)}^{\hbox{'}}{\left(M{\left({X}^{\prime }{\widehat{V}}^{-1}X\right)}^{-1}{M}^{\prime}\right)}^{-1}\left(M\widehat{\beta}\right)}{\mathit{\operatorname{rank}}(M)}\sim F\left(p,n-q\right),\mathrm{under}\ {H}_0 $$

$$ \mathrm{where}\ \widehat{V}=\widehat{Var\left(\widehat{\beta}\right)}={X}^{\prime }{\widehat{V}}_y^{-1}X\ \mathrm{and}\ {\widehat{V}}_y=\widehat{Var}(y). $$

We developed the above procedure to test association of multiple SNPs (genomic regions), which reduces the multiple-testing burden. However, this test can be seen as a single-marker association test that we have used to perform our GWAS study with appropriate multiple-testing correction.

## Results

**Fig. 1:**
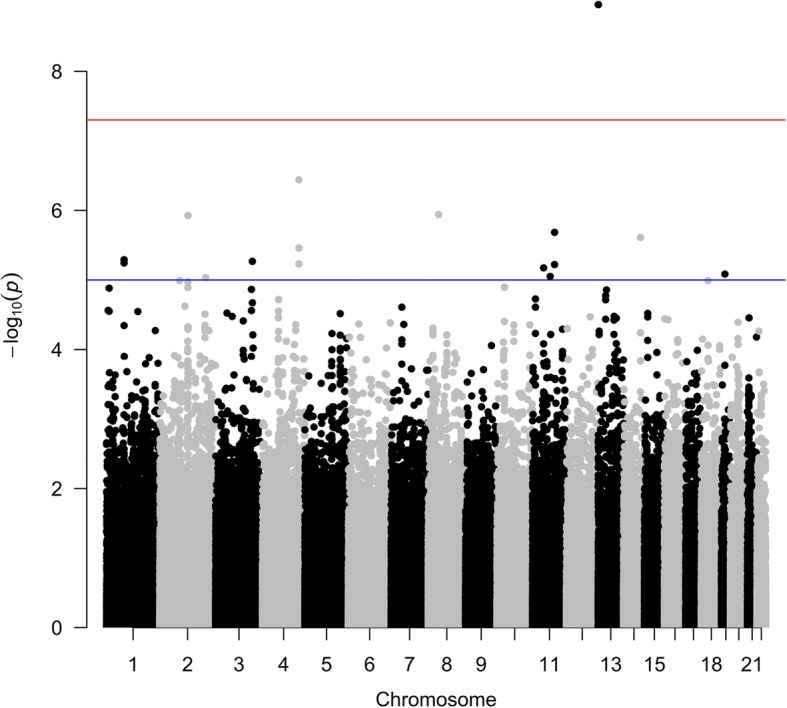
Manhattan plot of genome-wide *p* values of SNPs on interacting phenotypes, namely, TG and HDL

With longitudinal information using multiple phenotypes we identified only 1 significant SNP (Fig. 1) after BH correction. The SNP is rs2880301, located at *TPTE2* in an intron. rs2880301 is reported to be associated with HDL particle diameter and low-density lipoprotein (LDL) particle diameter [8] and is also known to confer protection against hepatocellular carcinoma [9]. However, we think that there might be other SNPs that remain unidentified as a consequence of small sample size. Hence we report the top 20 SNPs based on *p* value in Table 1. rs752273 is reported to be associated with cardiovascular diseases [10] while rs2896368 is known to be associated with α_1_-antitrypsin level [11].

**Table 1:** Top 20 SNPs associated with TG and HDL

Chr	SNPs	*p* Value	Base position
13	rs2880301	1.095660 × 10^*−* 9^	18,998,534
4	rs1909882	3.610927 × 10^*−* 7^	170,922,834
4	rs12510928	3.610927 × 10^*−* 7^	170,954,378
8	rs6558172	1.148044 × 10^*−*6^	22,547,997
2	rs752273	1.183976 × 10^*−*6^	108,297,537
11	rs1793368	2.066321 × 10^*−*6^	96,521,084
14	rs2896268	2.448163 × 10^*−*6^	93,935,461
13	rs2770297	2.707521 × 10^*−*6^	46,344,666
4	rs6835612	3.468092 × 10^*−*6^	170,955,790
1	rs4844913	5.117515 × 10^*−*6^	208,134,740
3	rs6440833	5.392711 × 10^*−*6^	154,128,934
1	rs924297	5.693772 × 10^*−*6^	76,996,366
4	rs13113929	5.873701 × 10^*−*6^	169,320,943
11	rs1255523	5.999600 × 10^*−*6^	95,019,155
11	rs395297	6.692577 × 10^*−*6^	37,093,845
19	rs4805303	8.219183 × 10^*−*6^	34,106,471
11	rs7929919	8.890781 × 10^*−*6^	78,529,128
2	rs2353319	9.293606 × 10^*−*6^	204,594,629
2	rs4676175	1.016761 × 10^*−* 5^	108,054,810
18	rs2419041	1.017765 × 10^*−* 5^	26,293,205

**Fig. 2:**
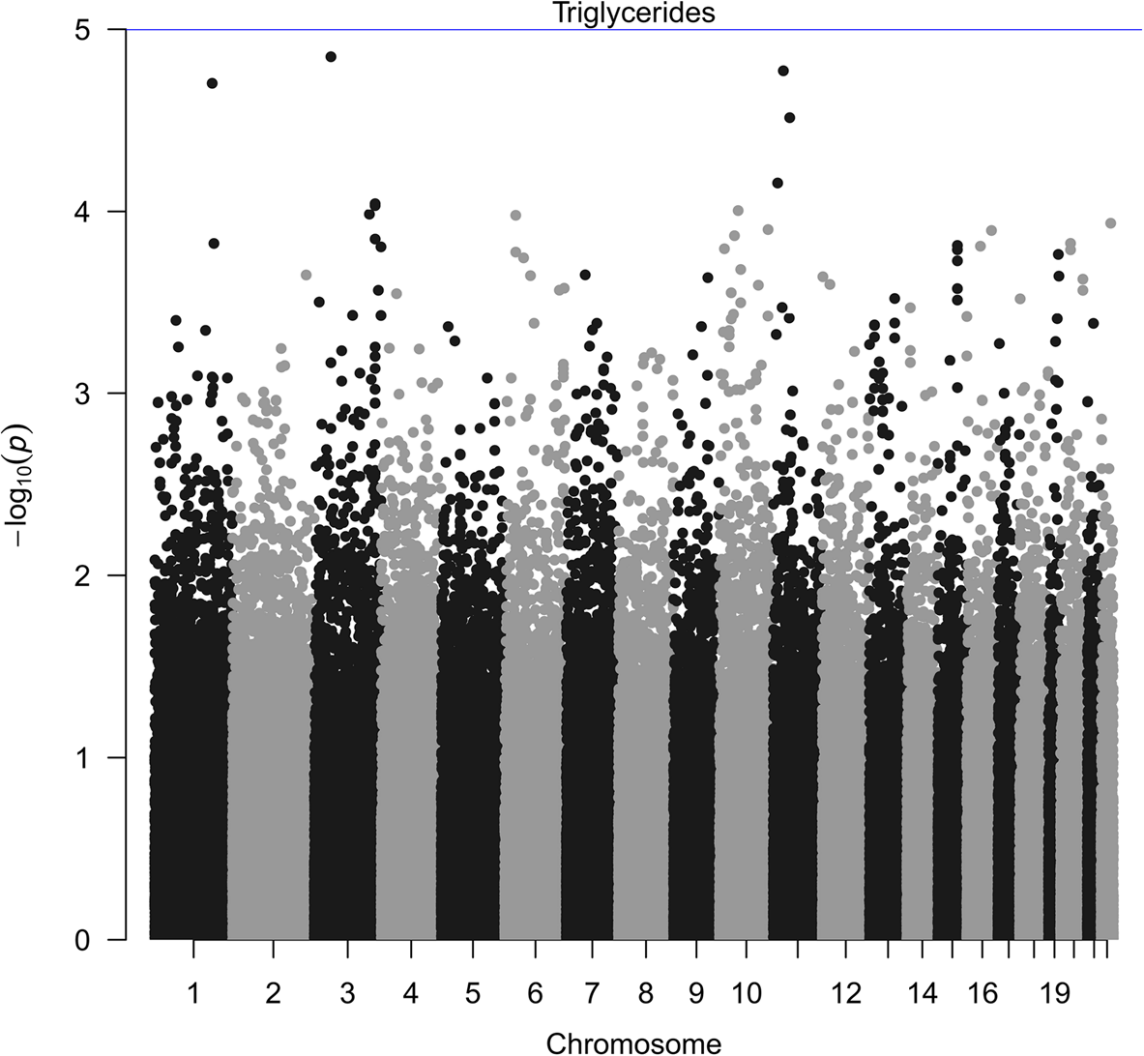
Manhattan plot of genome wide *p* values corresponding to response of drug treatment on TGs

To test the null hypothesis of no association with drug response, we examined 243,593 whole-genome SNPs and 18,266 genomic regions. The genomic regions in our study are (a) genic, that overlap the genotyped SNPs, and (b) intergenic, that lie between 2 consecutive genes and overlap the genotyped SNPs in the data set. After BH correction, none of the SNPs nor genomic regions showed significant association with the drug response (Fig. 2). However, we report the top 20 SNPs (Table 2) and top 20 genomic regions (Table 3). The moderately small sample size of the data compared to most of the GWAS might be a reason behind this result. Application of our methods to larger GWAS and further functional validation of the reported top loci might provide some directive for studying inflammatory biomarkers and response to fenofibrate treatment.

**Table 2:** Top 20 SNPs associated with fenofibrate treatment response

Chr	SNPs	*p* Value
1	rs7555566	1.982194 × 10^− 5^
1	rs6664332	1.499459 × 10^− 4^
3	rs1401072	1.416564 × 10^− 5^
3	rs13072632	9.056298 × 10^− 5^
3	rs11564450	9.281710 × 10^− 5^
3	rs1631395	1.034264 × 10^− 4^
3	rs9311268	1.418409 × 10^− 4^
3	rs6599150	1.418409 × 10^− 4^
6	rs13217251	1.048927 × 10^− 4^
10	rs2482583	9.882478 × 10^− 5^
10	rs3122227	1.257541 × 10^− 4^
10	rs7917347	1.358690 × 10^− 4^
11	rs2406928	1.692430 × 10^− 5^
11	rs10790087	3.064359 × 10^− 5^
11	rs1940088	6.968692 × 10^− 5^
15	rs17777266	1.537405 × 10^− 4^
16	rs7197943	1.272486 × 10^− 4^
20	rs6075087	1.497509 × 10^−4^
20	rs11087178	1.497509 × 10^− 4^
22	rs10427772	1.160345 × 10^− 4^

**Table 3:** Top 20 genomic regions associated with fenofibrate treatment

Chr	Genomic regions	*p* Value
1	*intergenic.snps CAPZB LOC105378614*	4.705439 × 10^*−* 4^
1	*intergenic.snps RCC2 ARHGEF10L*	6.471417 × 10^*−* 4^
2	*snps SCN1A*	6.896508 × 10^*−* 4^
3	*snps ABCC5*	1.877981 × 10^*−* 4^
3	*snps PQLC2L*	8.087247 × 10^*−* 4^
3	*snps IQCJ-SCHIP1*	8.150236 × 10^*−* 4^
4	*intergenic.snps C4orf33 LOC101927282*	2.817887 × 10^*−* 4^
5	*snps FAM81B*	4.593152 × 10^*−* 4^
5	*snps PKD2L2*	5.974084e × 10^*−* 4^
6	*intergenic.snps LOC105377967 CEP85L*	1.270536 × 10^*−* 4^
7	*intergenic.snps NUPL2 GPNMB*	7.784435 × 10^*−* 4^
8	*snps TRPA1*	4.326193 × 10^*−* 5^
9	*intergenic.snps TNFSF8 TNC*	1.822392 × 10^*−* 4^
9	*intergenic.snps HACD4 IFNB1*	2.491489 × 10^*−* 4^
9	*intergenic.snps DEC1 LOC101928775*	6.834450 × 10^*−* 4^
10	*intergenic.snps MAP3K8 LYZL2*	2.765323 × 10^*−* 4^
14	*snps NEMF*	3.979899 × 10^*−* 5^
18	*snps LO × HD1*	5.430775 × 10^*−* 4^
20	*snps LINC01524*	8.431111 × 10^*−* 5^
22	*intergenic.snps MIR4762 WNT7B*	4.120036 × 10^*−* 4^

## Discussion and conclusions

In this paper, we developed novel methods for (a) GWAS using longitudinal data and (b) GWAS/genomic region association with response to fenofibrate treatment based on a family-based design. These methods are agnostic to the choice of phenotype and can be generalized to any such study. Although we could not detect any novel biologically relevant locus that is significantly associated with response to fenofibrate treatment, we identified a few loci that are associated with TG and HDL levels. Our belief is that the primary reason for obtaining only a small number of significant association findings is the much smaller sample size in our analyses as compared to conventional GWAS. Validation in a larger sample might throw more light on the roles of the top few significantly associated SNPs and/or genomic regions in controlling TG and HDL levels. Nevertheless, this study emphasizes the effect of administering fenofibrate to individuals with specific genetic profiles.

We pruned the available set of SNPs to an independent set of SNPs in our GWAS, primarily to reduce the multiple-testing burden. Because many studies impute SNPs, and hence augment the number of available SNPs, to explore association findings for previously reported SNPs that have not been genotyped, our strategy has a caveat in the sense of reduction in the overall power of the GWAS. Similarly, while our proposed method involves simultaneous testing of multiple SNPs within a gene in order to evaluate association at the gene level, it may yield lower powers compared to the usual single SNP analyses in GWAS.

We imputed the missing phenotype data using a known heritability value [5] and have applied the EM algorithm. Although studies show that such imputation may lead to some loss of power and hence seems to be a limitation of our current method, the intuition behind the imputation strategy was to use the maximum phenotype data in our analyses. A more general model that includes the genotype data can be developed in a likelihood framework for testing association, but this would increase substantial computational complexity while calculating the test statistic.

Association findings based on any real data set are susceptible to being false positives. If these findings validate previous reports of association, they are more likely to be true positives. In case of novel significant findings, it is necessary to either validate them in an independent data set or, alternatively, to perform extensive simulations under similar genotype and phenotype structures to evaluate the false-positive rates of the underlying test procedures.
